# Dysregulation of microRNAs and target genes networks in human abdominal aortic aneurysm tissues

**DOI:** 10.1371/journal.pone.0222782

**Published:** 2019-09-20

**Authors:** Neire Niara Ferreira de Araujo, Hui Tzu Lin-Wang, Juliana de Freitas Germano, Pedro Silvio Farsky, Andre Feldman, Fabio Henrique Rossi, Nilo Mitsuru Izukawa, Maria de Lourdes Higuchi, Felicio Savioli Neto, Mario Hiroyuki Hirata, Marcelo Chiara Bertolami

**Affiliations:** 1 Department of Clinical Cardiology, Dante Pazzanese Institute of Cardiology, Sao Paulo, Brazil; 2 Laboratory of Molecular Investigation in Cardiology, Dante Pazzanese Institute of Cardiology, Sao Paulo, Brazil; 3 School of Pharmaceutical Sciences, University of Sao Paulo, Sao Paulo, Brazil; 4 Department of Vascular Surgery, Dante Pazzanese Institute of Cardiology, Sao Paulo, Brazil; 5 Laboratory of Cardiac Pathology, Heart Institute, School of Medicine, University of Sao Paulo, Sao Paulo, Brazil; National Institutes of Health, UNITED STATES

## Abstract

**Background:**

Abdominal aortic aneurysm (AAA) is a pathological enlargement of infrarenal aorta close to the aortic bifurcation, and it is an important cause of mortality in the elderly. Therefore, the biomarker identification for early diagnosis is of great interest for clinical benefit. It is known that microRNAs (miRNAs) have important roles via target genes regulation in many diseases. This study aimed to identify miRNAs and their target genes involved in the pathogenesis of AAA.

**Methods:**

Tissue samples were obtained from patients who underwent AAA surgery and from organ donors (control group). Quantitative PCR Array was applied to assess 84 genes and 384 miRNAs aiming to identify differentially expressed targets (AAA n = 6, control n = 6), followed by validation in a new cohort (AAA n = 18, control n = 6) by regular qPCR. The functional interaction between validated miRNAs and target genes was performed by the Ingenuity Pathway Analysis (IPA) software.

**Results:**

The screening cohort assessed by PCR array identified 10 genes and 59 miRNAs differentially expressed (≥2-fold change, p<0.05). Among these, IPA identified 5 genes and 9 miRNAs with paired interaction. *ALOX5*, *PTGIS*, *CX3CL1* genes, and miR-193a-3p, 125b-5p, 150-5p maintained a statistical significance in the validation cohort. IPA analysis based on the validated genes and miRNAs revealed that eicosanoid and metalloproteinase/TIMP synthesis are potentially involved in AAA.

**Conclusion:**

Paired interactions of differentially expressed *ALOX5*, *PTGIS*, *CX3CL1* genes, and miR-193b-3p, 125b-5p, 150-5p revealed a potentially significant role of the eicosanoid synthesis and metalloproteinase/TIMP pathways in the AAA pathogenesis.

## Introduction

Abdominal aortic aneurysm (AAA) is a pathological enlargement of infrarenal aorta close to the aortic bifurcation, and it is an important cause of mortality in the elderly. The AAA incidence in individuals older than 60 years of age is higher in men than in women; 5% vs. 1%, respectively [1,2]. Smoking is the main risk factor for the expansion of AAA [3] and other risks include age, male gender, lower HDL cholesterol levels, and genetic susceptibility [4,5].

Ultrasonography (US) is routinely used to diagnose and monitoring AAA, with sensitivity and specificity close to 100% in the late stage of the disease; most cases are detected by chance during routine abdominal US [6,7]. Aortic diameter larger than 3 cm is suggestive of AAA; aortic diameter larger than 5 cm shows worse outcome, requiring intense monitoring [8] and treatment to reduce aortic inflammation, proteolysis, and support vascular smooth muscle cell recovery [6]. The annual rupture risk increases with aortic enlargement, reaching 30% in AAA larger than 7 cm [9]. In these cases, surgical repair is advised; however, preoperative risk also has to be taken into consideration.

The mechanisms underlying AAA are complex and not completely understood. However, some studies suggest that AAA development is mainly associated with increased biomechanical stress, proteolytic degeneration of elastin and collagen in the aortic wall, inflammation processes, genetic factors, and immune responses [10,11]. Some molecules, such as *CCL22* [12] and *CTLA4* [13] are suggested as biomarkers for AAA diagnosis.

Adequate regulation of gene expression is critical for all cellular processes and its dysregulation may contribute to disease. Gene expression can be regulated at the post-transcriptional level by small non-coding RNAs, known as microRNAs (miRNA), which are short, single-stranded RNAs (approximately 22 bp) that control mRNA stability or translational repression via base pairing with regions within the 3' untranslated region of the target RNA [14]. The miRNAs are found not only inside the cells, but also in biological fluids such as serum, plasma, urine, and saliva [15], and can be delivered into different tissues through vesicles and lipoprotein transport [16]. The role and implications of miRNAs in animal models and their relevance to human AAA have also been discussed [17]. The circulating miR-455-3p has been suggested as a promising biomarker for the early diagnosis of AAA [18]. However, the role of miRNAs in AAA development remains to be clarified.

To date, the available treatments for AAA are surgical or interventional using different kinds of prosthesis, and new targets need to be identified in order to bring alternatives to diagnosis and therapy. Here, we analyzed miRNA and mRNA profiles of human AAA tissues and applied miRNA-mRNA interactions to predict molecular pathways that may outline potential targets for therapeutic approaches to control AAA progression.

## Materials and methods

### Ethics statement

This study was approved by the ethics committee of the Dante Pazzanese Institute of Cardiology in Sao Paulo, Brazil (number 1470/2014). Each participant signed an informed consent after receiving a verbal explanation of the study. Patients who had cognitive deficit were not enrolled. The donation term was signed by responsible relatives in accordance with the Brazilian laws for organ and tissue donation.

### Abdominal aortic tissues samples

Abdominal aortic tissues were obtained from patients undergoing AAA repair surgery and from brain-dead but heart beating organ donors. The characteristics of the screening and validation cohort are shown in Table 1. The excised segments of the abdominal aorta were collected in RNAlater^®^ stabilization solution (Ambion Inc., MN, USA) and stored at -20 °C until assayed.

**Table 1:** Biodemographic characteristics of screening and validation cohort.

Variable	Screening Cohort			Validation Cohort		
	AAA (n = 6)	OD (n = 6)	p-value	AAA (n = 18)	OD (n = 6)	p-value
Age (year, mean ± SD)	68.8 (± 5.5)	42.8 (± 9.3)	<0.001	71.0 (± 5.6)	49.5 (± 5.1)	<0.001
Male (n,%)	5 (83,3)	3 (50)	0.545	14 (77.8)	2 (33.3)	<0.001
Ever smoker (n,%)	5 (83,3)	2 (33.3)	0.242	16 (88.9)	5 (83.3)	0.422
Alcohol consumption (n,%)	3 (50)	2 (33.3)	1.000	5 (27.8)	2 (33.3)	0.441
Hypertension (n,%)	5 (83.3)	4 (66.7)	1.000	16 (88.9)	4 (66.7)	<0.001
Dyslipidemia (n,%)	5 (83.3)	0	<0.05	10 (55.5)	0	<0.05
Diabetes (n,%)	2 (33.3)	0	<0.05	3 (16.7)	0	<0.05
Family history of CAD (n,%)	2 (33.3)	0	<0.05	2 (11.1)	0	<0.05
aneurysm diameter (cm, mean ± SD)	5.6 (± 0.89)	<3.0	<0.05	5.9 (± 1.27)	<3.0	<0.05

AAA: Abdominal aortic aneurysm; OD: organ donors; CAD: coronary artery disease

Data are shown as average and standard deviation for numeric variables and as count and percentage for categorical variables. The t-test was performed to compare numeric variables and Fisher’s exact test was performed to compare categorical variables.

To check if the tissues from organ donors were adequate to use as a control, we applied Verhoeff´s histological staining to assess elastin, an extracellular matrix protein. The image was acquired by a Scanscope CS system unit (Aperio Technologies Inc., CA, USA), with an Olympus UPlanSApo objective lens, with specifications of 20x magnification power and 0.75 numerical aperture attached to the scanner. Images were analyzed using the Aperio program ImageScope View software (Aperio Technologies, Inc., CA, USA). Results were reported as the positive staining percentage area per total tissue area.

### RNA extraction and qPCR array analysis

Tissue samples from AAA (n = 6) and control group (n = 6) were randomly selected for qPCR array analysis.

To obtain mRNA, 20 mg of aortic tissues containing all layers were firstly homogenized by TissueRuptor^®^ (Qiagen, GmbH, Hilden, Germany), followed by extraction using RNeasy Microarray Tissue Mini Kit (Qiagen, GmbH, Hilden, Germany), according to manufacturer recommendations. RNA quantification was determined using a Qubit^®^ spectrophotometer system (Life Technologies, Foster City, CA, USA) and sample quality was assessed by Agilent Technologies 2200 Tape Station (Agilent Technologies Inc., USA). All samples had RNA integrity number (RIN) higher than eight, therefore, they were suitable for downstream processing.

The RT^2^ First Strand Kit (Qiagen, GmbH, Hilden, Germany) was used for cDNA synthesis and quality control was performed using RT2 RNA QC RT2 Profiler PCR Array plates (PAHS-999Z), which looked out for the presence of reverse transcription inhibitors, PCR amplification efficiency, and DNA contamination.

The screening of candidate genes involved in AAA pathogenesis was performed by the RotorGene^®^ Real-Time PCR system (Qiagen, Hilden, Germany) using RT2 Profiler PCR Arrays (PAHS-015Z, Qiagen, Hilden, Germany). This array includes 84 genes involved in angiogenesis, vasoconstriction, vasodilation, inflammatory response, apoptosis, cell adhesion molecules, coagulation, and platelet activation.

The PCR cycling condition was set as follows: 1 cycle at 95 °C for 10 min, 40 cycles at 95 °C for 15 s, and 60 °C for 30 s. The Qiagen web-based PCR array data analysis software was applied to obtain fold change of gene expression. Genes showing ≥2-fold change and p-value <0.05 were considered as differentially expressed. A cycle of threshold (Ct) value higher than 35 was considered as no amplification.

### MicroRNA extraction and qPCR array analysis

The total RNA including miRNAs were isolated using RNeasy Fibrous Tissue Mini Kit (Qiagen, GmbH, Germany) according to the protocol. RNA was quantified using the RNA assay kit in Qubit^®^ spectrophotometer. All samples showed RIN (RNA integrity number) >8.0 and were suitable for downstream processing. An input of 800ng of total RNA was used to prepare the cDNA following the miScript II RT protocol (Qiagen, Hilden, Germany). The quality control of cDNA samples was assessed by miScript miRNA QC PCR plates (MIHS-989ZE, Qiagen, Hilden, Germany). Quantitative PCR was performed according to manual instructions in QuantiStudio^®^ 12k Flex Real-Time PCR system (Thermo Fisher Scientific, Waltham, MA, USA) using Human miFinder 384HC miScript miRNA PCR Arrays (Qiagen, Hilden, Germany). The PCR cycling conditions were set as follows: 1 cycle at 95 °C for 15 min; 40 cycles of 3 step cycling: 94 °C for 15 s, 55 °C for 30 s and 70 °C for 30 s. The Ct values were submitted to the Qiagen web-based PCR array data analysis software and the 2^-ΔΔCt^ formula was used to compare miRNA expression between groups. The global mean normalization method was used to normalize the miRNA Cts [19]. The miRNAs showing ≥ 2-fold change and p-value < 0.05 were considered as differentially expressed. Ct values higher than 35 were not included in the analysis.

### Ingenuity Pathway Analysis^®^ (IPA)

Functional analysis was performed by IPA^®^ software (Qiagen, Hilden, Germany). Interactions between differentially expressed miRNAs and target genes were assessed by opposite pairwise analysis (Table 2), and Molecular Activation Prediction (MAP) analysis was applied to predict molecular activation or inhibition that may be involved in AAA pathogenesis. New pathway analysis was performed to investigate the interaction between the validated genes and miRNAs. Intermediary molecules were added to the pathway to clarify the link between these miRNAs and their target genes and explain the relevant roles in the AAA context.

**Table 2:** Differently expressed miRNA was paired up with the respective target gene by IPA pairing analysis.

miRNA	Fold change	p-value	Target genes	Fold change	p-value
hsa-miRNA-1207-5p	9.3	0.037	CX3CL1	-5.8	0.017
hsa-miRNA-125b-5p	-3.5	0.005	ALOX5	2.8	0.011
hsa-miRNA-150-5p	7.5	0.007	PTGIS	-8.7	0.031
hsa-miRNA-16-5p	2.2	0.040	CX3CL1	-5.1	0.017
hsa-miRNA-182-5p	3.3	0.013	FN1	-2.9	0.041
hsa-miRNA-193b-3p	-3.1	0.008	ALOX5	2.8	0.011
hsa-miRNA-200c-3p	2.1	0.015	FN1	-2.9	0.041
hsa-miRNA-34c-5p	2.5	0.026	AGTR1	-2.6	0.022
hsa-miRNA-34c-5p	2.5	0.026	PTGIS	-8.7	0.031
hsa-miRNA-34b-3p	2.1	0.045	FN1	-2.9	0.041

### Validation of paired expressed genes and miRNAs

The validation cohort consisted of other samples different from the ones employed for screening, 18 AAA samples, and 6 organ donor control.

All samples were tested for miRNAs and genes that showed paired interactions in the paired expression analysis (Table 2). The miRNA and mRNA analysis followed the same steps described above using the RT^2^ qPCR primer assays for gene expression and miScript primer assays for miRNA expression. Assays used for this proposal were: *ALOX5* (#PPH2590G), *CX3CL1* (#PPH689C), *PTGIS* (#PPH2582A), *FN1* (#PPH143B), *AGTR1* (#PPH2362F), TIMP1 (#PPH00771C), miR-1207-5p (#MS14189), miR-125b-5p (#MS6629), miR-150-5p (#MS3577), miR-16-5p (#MS31493), miR-193b-3p (#MS31549), miR-34c-5p (#MS3332). The average expression of three reference genes: *ACTB* (#PPH73G), *B2M* (#PPH1094E) and *GAPDH* (#PPH150F) were applied for normalization of the gene expression, and in case of miRNAs expression, the average expression of SNORD61 (#MS33705), SNORD68 (#MS33712) and RNU6-6p (#MS33740) was applied for normalization.

The relative expression was calculated by the 2^-ΔΔCt^ formula [20], and results were reported as fold change.

### Statistical analysis

The biodemographic data was reported as average and standard deviation or as count and percentage. The t-test was used to compare numerical variables from the biodemographic data and the likelihood ratio or Fisher’s exact test was used to compare categorical variables. The elastin detected by immunohistochemical staining, as well as the miRNA and gene expression were analyzed by a Mann-Whitney U test for non-parametric data and reported as median, 25^th^ and 75^th^ percentiles. Differences were considered statistically significant when p < 0.05.

## Results

### Biodemographic data and sample characteristics

As expected, patients from the AAA group were older than the donors. Also, the AAA group showed a higher frequency of male sex, hypertension, dyslipidemia, diabetes and family history of coronary artery disease (Table 1).

The aneurysm from the AAA group achieved a 5.9 cm diameter on average while the control group had an abdominal aortic diameter smaller than 3.0 cm detected by observational analysis only. Comparative microscopic analysis revealed that AAA tissues had a significant decrease in elastin fiber. The 25^th^ and 75^th^ percentile of elastin detected by Verhoeff´s staining was 1.0% and 9.3% in AAA compared to 3.1% and 24.6% in the control group (p < 0.001), respectively.

### Gene and miRNA qPCR array analysis

Ten out of 84 genes analyzed by RT-qPCR were differentially expressed in the AAA group (n = 6) when compared to the control group (n = 6). Six genes (*PTGIS*, *CX3CL1*, *ITGB1*, *COL18A1*, *FN1*, and *AGTR1*) were significantly downregulated and four other genes (*SPHK1*, *TYMP*, *ALOX5*, *HIF1A*) were significantly up-regulated (Table 3).

**Table 3:** Differently expressed genes were identified by PCR array: AAA (n = 6) compared to organ donor control (n = 6).

Gene’s symbol	Ref. Sequence	Gene’s description	Folde Change	Regulation	p-value
SPHK1	NM_021972	Sphingosine kinase 1	5.9	up	0.008
TYMP	NM_001953	Thymidine phosphorylase	3.4	up	0.012
ALOX5	NM_000698	Arachidonate 5-lipoxygenase	2.8	up	0.011
HIF1A	NM_001530	Hypoxia inducible factor 1, alpha subunit	2.3	up	0.044
PTGIS	NM_000961	Prostaglandin I2 (prostacyclin) synthase	-8.8	down	0.031
CX3CL1	NM_002996	Chemokine (C-X3-C motif) ligand 1	-5.1	down	0.017
ITGB1	NM_002211	Integrin, beta 1 (fibronectin receptor)	-4.2	down	0.038
COL18A1	NM_030582	Collagen, type XVIII, alpha 1	-3.2	down	0.013
FN1	NM_002026	Fibronectin 1	-2.9	down	0.041
AGTR1	NM_031850	Angiotensin II receptor, type 1	-2.6	down	0.022

AAA: abdominal aortic aneurysm

The expression analysis of 372 miRNAs by qPCR array showed a total of 59 differentially expressed miRNAs in the AAA group, from which 35 were down-regulated and 24 were up-regulated (Table 4).

**Table 4:** Differently expressed miRNAs were identified by PCR array: AAA (n = 6) compared to organ donor control (n = 6).

Mature miRNA	up-regulation	p-value	Mature miRNA	down-regulation	p-value
hsa-let-7g-3p	9.85	0.014	hsa-let-7a-5p	-2.02	0.023
hsa-miR-1207-5p	9.27	0.036	hsa-let-7c-5p	-2.14	0.002
hsa-miR-142-3p	7.2	0.033	hsa-miR-100-5p	-2.75	0.003
hsa-miR-144-5p	5.54	0.003	hsa-miR-125a-5p	-2.7	0.015
hsa-miR-150-5p	7.5	0.007	hsa-miR-125b-5p	-3.52	0.005
hsa-miR-155-3p	3.54	0.011	hsa-miR-133a-3p	-9.33	0.029
hsa-miR-15b-3p	4.58	0.003	hsa-miR-133b	-7.95	0.009
hsa-miR-16-5p	2.16	0.040	hsa-miR-140-3p	-4.55	0.001
hsa-miR-181a-3p	4.58	0.007	hsa-miR-140-5p	-2.96	0.008
hsa-miR-182-5p	3.34	0.013	hsa-miR-143-3p	-7.31	0.001
hsa-miR-200c-3p	2.08	0.015	hsa-miR-143-5p	-9.2	0.001
hsa-miR-212-3p	2.09	0.047	hsa-miR-145-5p	-7.15	0.008
hsa-miR-29a-5p	2.98	0.005	hsa-miR-145-3p	-5.42	0.001
hsa-miR-31-5p	6.08	0.018	hsa-miR-193a-3p	-2.58	0.038
hsa-miR-338-3p	4.87	0.033	hsa-miR-193a-5p	-3.12	0.001
hsa-miR-34b-3p	2.05	0.045	hsa-miR-193b-3p	-3.11	0.008
hsa-miR-34c-5p	2.53	0.026	hsa-miR-193b-5p	-2.51	0.002
hsa-miR-363-3p	3.28	0.024	hsa-miR-196b-5p	-4.73	0.006
hsa-miR-378a-3p	2.05	0.003	hsa-miR-23a-3p	-2.07	0.006
hsa-miR-378a-5p	2.19	0.006	hsa-miR-23b-3p	-20.83	0.001
hsa-miR-425-5p	2.32	0.013	hsa-miR-23b-5p	-2.67	0.019
hsa-miR-451a	5.61	0.010	hsa-miR-24-3p	-2.31	0.001
hsa-miR-454-3p	3.09	0.010	hsa-miR-27b-3p	-3.67	0.001
hsa-miR-542-5p	2.62	0.008	hsa-miR-27b-5p	-5.24	0.001
			hsa-miR-28-3p	-2.32	0.004
			hsa-miR-28-5p	-2.15	0.002
			hsa-miR-29b-2-5p	-2.69	0.001
			hsa-miR-328-3p	-3.78	0.037
			hsa-miR-331-5p	-4.52	0.001
			hsa-miR-365b-3p	-3.11	0.005
			hsa-miR-504-5p	-5.96	0.004
			hsa-miR-574-3p	-2.16	0.008
			hsa-miR-744-5p	-2.62	0.010
			hsa-miR-99a-3p	-2.8	0.032
			hsa-miR-99b-5p	-3.22	0.042

### Pathway analysis and qPCR validation

After identifying differentially expressed genes and miRNAs in the AAA group, the IPA software was used to recognize paired interactions between miRNAs and their target genes. Ten paired interactions were identified, which involved five differentially expressed genes and 9 miRNAs (Table 2).

**Fig 1:**
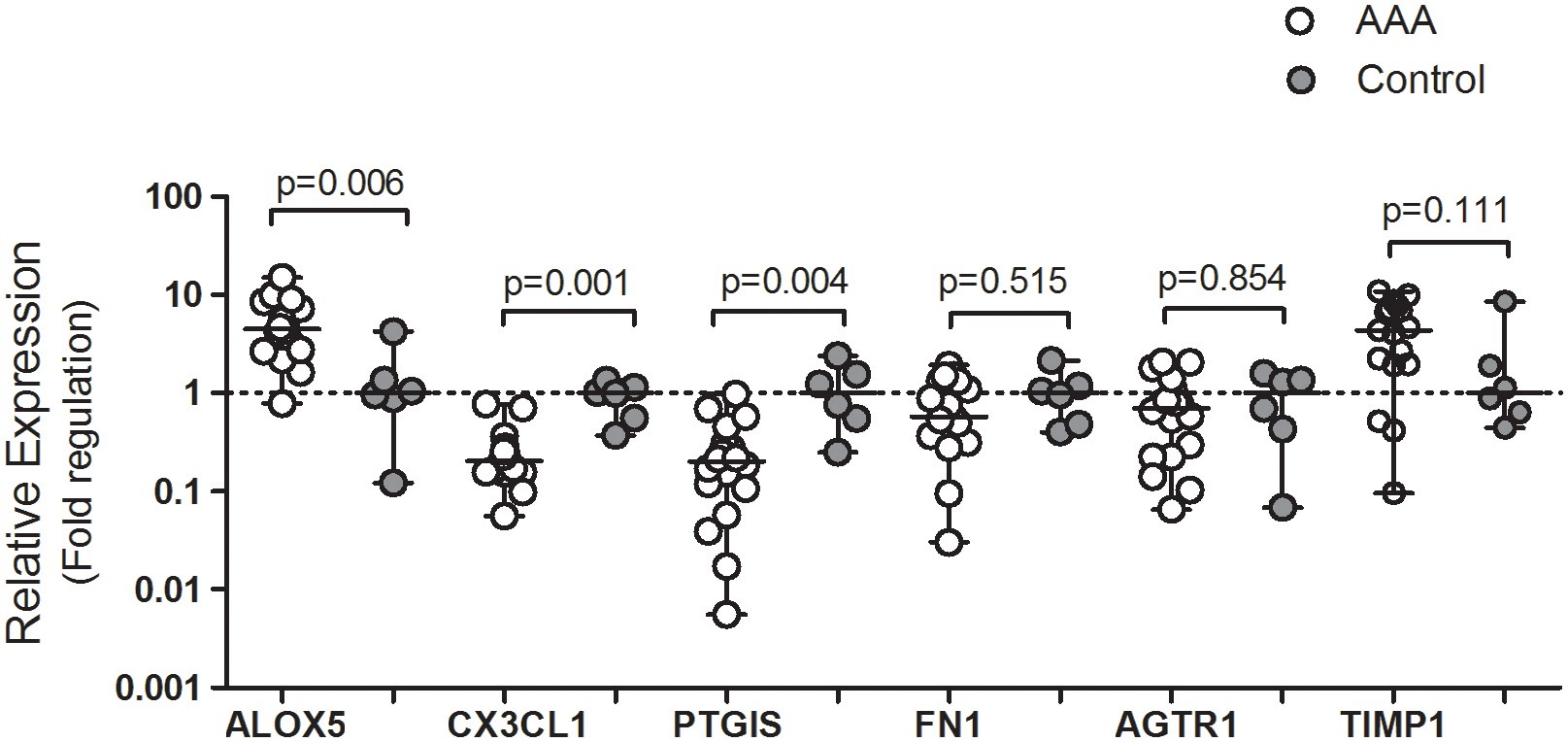
Validation of gene expression by qPCR. The relative expression was calculated using the 2^-ΔΔCt^ formula and Mann-Whitney test was performed to compare AAA (n = 18) and control groups (n = 6). The null hypothesis was rejected if the p-value was lower than 0.05.

**Fig 2:**
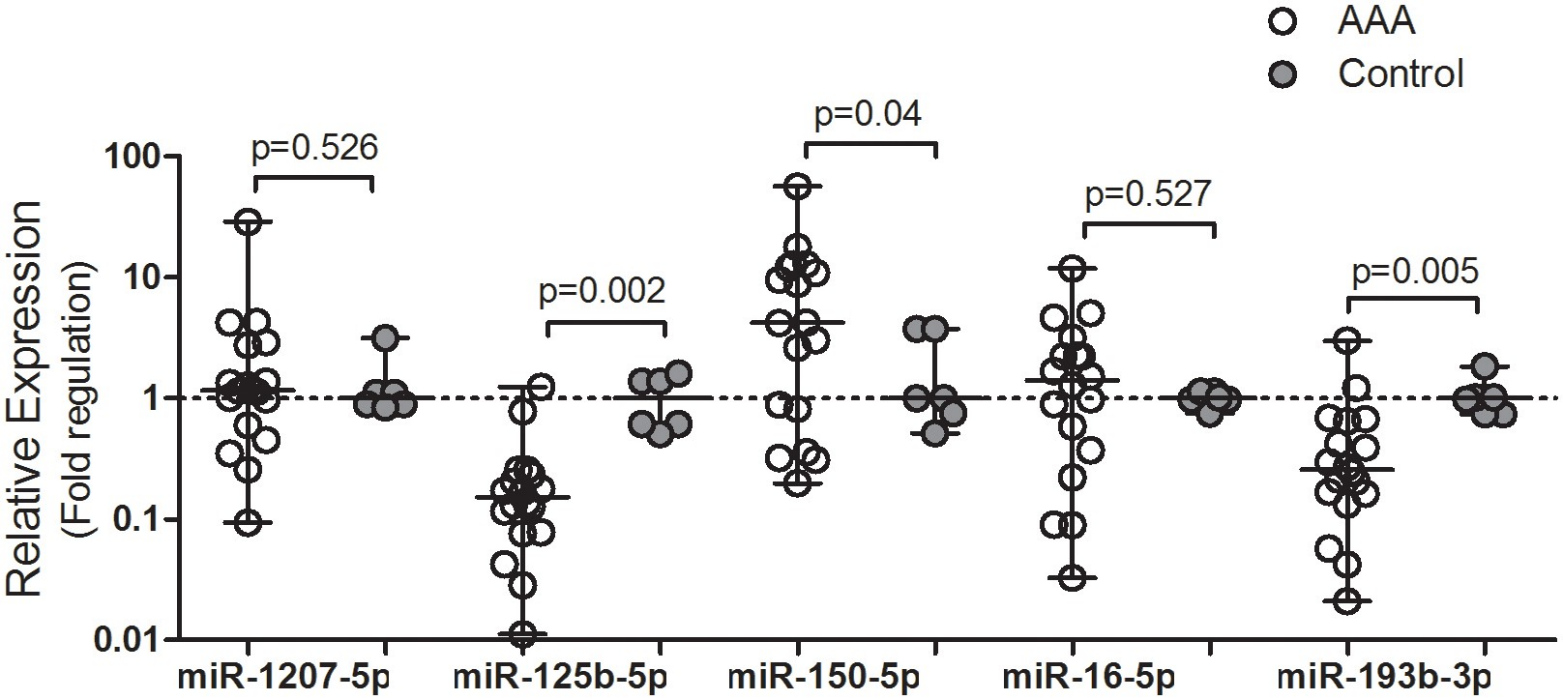
Validation of microRNA expression by qPCR. The 2^-ΔΔCt^ formula was applied to obtain relative expression and Mann-Whitney test was performed to compare AAA (n = 18) and control group (n = 6). The null hypothesis was rejected if the p-value waslower than 0.05.

**Fig 3:**
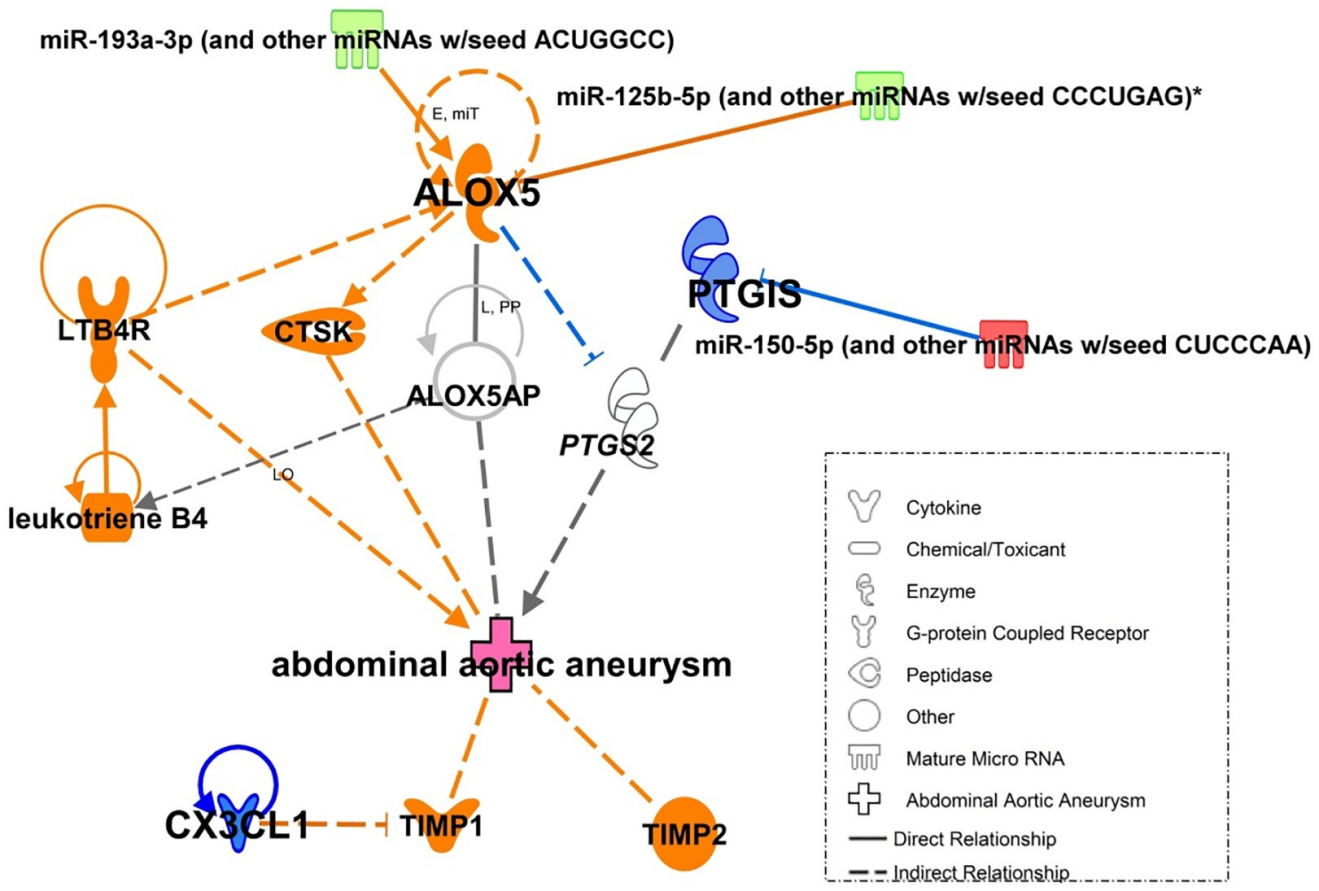
Ingenuity Pathway Analysis (IPA) showed predicted interactions between validated miRNAs and their respective targets genes in abdominal aortic aneurysm. Intermediary molecules are also showed in grey to clarify the connections of miRNAs and targets. The blue lines and molecules represent inhibition; orange lines and molecules represent activation; green represents miRNA down-regulation, and red represents miRNA up-regulation.

Three out of six genes (*FN1*, TIMP1 and *AGTR1*) lost statistical relevance in the validation cohort, and, consequently, miRNAs associated with these genes (miR-182-5p, 200c-3p, and 34b-3p) did not follow validation. It is worth mentioning that 12 out of 18 samples had upregulation of TIMP1 ranging from 2 to 10 folds, even though statistical difference was not detected (Fig 1). Six miRNAs (miR-1207-5p, 125b-5p, 150-5p, 16-5p, 193b-3p and 34c-5p) showed paired interactions with genes and followed validation (Fig 2). Several samples showed miR-34c-5p Ct higher than 35, indicating no detectable expression. This miRNA was, therefore, excluded from the IPA representation on Fig 3. Although miRNAs 1207-5p and 16-5p are highly upregulated in array data, they showed no differences in the RT-PCR validation cohort and, were not included in the final IPA network.

Through bioinformatic analysis, the results showed that eicosanoid synthesis (arachidonic acid and leukotriene) and metalloprotease/TIMP pathway are possibly the two main pathways involved in AAA pathogenesis that are directly regulated by differentially expressed miRNAs (Fig 3).

## Discussion

There is some literature showing the assess of miRNA or mRNA profile in AAA tissues [13,21]. However, the identified target substantially varies among studies, probably because different aortic fragments were assessed and distinct methods were employed. It is known that each aortic segment has a different embryonic origin and shows vascular smooth muscle diversity [22]; therefore, gene expression profile may also vary between aortic segments. In this study, we carefully obtained samples from the same aortic infra-renal fragments for both groups (AAA vs control). The AAA average aortic diameter was 5.9 cm, and there was a significant elastin fiber degradation associated with collagen down-expression, which characterizes chronic aneurysm. It is important to mention that this study was not designed to evaluate early AAA, once the tissues were obtained in a later stage, concomitant with the surgery. These results, combinedwith a serum miRNAs analysis [18], might be interesting to identify biomarkers for early AAA diagnosis.

According to Pahl and colleagues, target genes associated with apoptosis and activation of T cells are mainly involved in AAA. However, other vascular cells may also probably contribute to AAA pathogenesis [21]. For this proposal, firstly, we evaluated the expression of 84 genes associated with vascular function, and 372 most abundantly expressed miRNAs and best-characterized miRNAs in miRBase (www.miRBase.org). The screened genes and miRNAs were validated in a new cohort, consisted of 18 AAA samples. Surprisingly, two genes (*FN1* and *AGTR1*) and three miRNAs (16-5p, 1207-5p, and 34c-5p) previously screened by PCR array lost the statistical significance in the validation test. Probably, the number of samples assessed in the array and the tissue heterogeneity contributed to the difference between these results, reinforcing the importance of validation in array approaches. Tavares et. al. showed that even though the biomechanical resistance of AAA varies with size or location of aorta, no histological or histochemical differences were detected in situ [23], hence the expression of gene and miRNA could be more sensitive for AAA monitoring.

Based on genes and miRNAs identified in the validation study, the IPA analysis showed two pathways associated with AAA: the eicosanoid synthesis pathway, with *ALOX5* and *PTGIS* genes participation, and metalloprotease/TIMP pathway, which involves the *CX3CL1* gene.

The role of *ALOX5* in the eicosanoid synthesis pathway is well-established. This gene encodes the enzyme arachidonate 5-lipoxygenase which, in association with ALOX5AP protein [24], to catalyze the conversion of 5-HPETE to leukotriene A4 (LTA4), favor the proinflammatory leukotrienes biosynthesis [25] and increases the infiltration of immune cells through leukotriene B4 signaling [26]. ALOX5 also plays a key role in cathepsin K (CTSK) modulation [27], which is directly involved in AAA formation through mechanisms such as collagen turnover [28], T-cell proliferation, and smooth muscle cell apoptosis [29]. The regulation of *ALOX5* transcript by miRNA-125b-5p was previously reported in several cell lines [30]. In this study, it was noted a down-regulation of miRNA-125b-5p and miR-193a-3p in AAA tissues leading to up-regulation of *ALOX5* gene, which results in a leukotriene production increase and empowers aortic wall inflammation and injury.

Another gene involved in the eicosanoid synthesis pathway identified in our studyis *PTGIS*. This gene encodes prostaglandin I2 synthase and catalyzes prostacyclin (PGI2) synthesis from prostaglandin H2. PGI2 is widely distributed and predominantly found in vascular endothelial and smooth muscle cells and it has an inhibitory role in platelet aggregation and thrombus formation [31]. An imbalance of prostacyclin and its physiological antagonist thromboxane A2 (TXA2) contributes to the development of myocardial infarction, stroke, and atherosclerosis [32,33]. The frequencies of *PTGIS* (rs5602CC) and *PTGS2* (rs20417CC) variants were significantly higher in patients with carotid plaque compared with patients without plaque, and are associated with carotid plaque vulnerability, platelet activation and TXA2 levels in ischemic stroke patients [34]. Our data suggests that up-regulation of miRNA-150-5p is possibly repressing *PTGIS* expression, which indirectly contributed to AAA progression through the leukotriene pathway. In this study, we did not assess *PTGS2* expression; but it is known that *PTGS2* is an induced form of prostaglandin-endoperoxide synthase, also known as cyclooxygenase, which is the key enzyme in prostaglandin biosynthesis. High-level tumor miR-21 expression may potentiate the PTGS2/PGE2 pathway and suppress antitumor immunity [35]. In AAA, miR-21 is usually upregulated [11,36], and modulation of miR-21 expression can limit AAA expansion through the inhibition of the expression of the phosphatase and tensin homolog (PTEN) protein [37]. In our array, the results showed that miR-21-3p is 2.6 folds higher in AAA than control, but the difference was not statistical significant (p = 0.210). Further study can clarify if miR-21 also regulates PTGS2/PGE2 pathway in AAA context.

The down-regulation of *CX3CL1* (chemokine *C-X3-C* motif ligand 1) gene in AAA tissue was also observed in validated cohort. The protein codified by this gene inhibits tissue inhibitors of metalloproteinase (TIMP) and controls the turnover of matrix metalloproteinase (MMP). Recently, Ma et. al. described that the overexpression of miR-195 may induce the protein expression of MMP-2 and MMP-9 in AAA [38]. However, our study showed that miR-195 is equally expressed between AAA and control groups and,consequently, it was not included in the validation study. Through IPA^®^ bioinformatic analysis, we observed down-regulation of *CX3CL1* that favors *TIMP1* gene activation. Furthermore, in validation cohort, 12 out of 18 samples showed upregulation of TIMP1 ranging from 2 to 10 folds. Together, *TIMP1* and *TIMP2* suppress the expression of MMP. These results corroborate the existing literature showing the increase of TIMP expression in AAA [39]. However, other authors reported opposite findings [40]. Probably different models used by these studies could explain these conflicting results. *CX3CL1*, also known as fractalkine, chemoattracts T cells and monocytes, and their adhesive and migratory functions by interacting with the receptor CX3CR1 on endothelial cells [41]. We could not validate the expression of miRNAs 16-5p and 1207-5p, but we do not discard the involvement of an additional miRNA not presented in the array panel in the down-regulation of *CX3CL1* gene, or possible indirect regulation.

Our results identified *SPHK1* as the most upregulated gene in AAA. Although no information in the literature supports the role of this gene in the development of AAA, it is known that *SPHK1* regulates endothelial function [42], and could indirectly be involved in AAA pathogenesis.

Although it was not included in the validation step, it is worth mentioning that miRNA-23b-3p is by far the most expressed miRNA identified in this study; its expression in AAA tissue was almost 21-fold lower than that in the normal aorta. It has been shown that the miRNA-23b-3p plays a critical role in a wide range of biological processes, including angiogenesis [43] and positively regulating cardiac hypertrophy [44]. Its downregulation induces phenotypic switching of vascular smooth muscle cells from a low proliferative to a highly proliferative phenotype [45]. In this study, the IPA^®^ bioinformatic tools did not identify targets among the differentially expressed genes in our PCR array. As previously mentioned, each miRNA can regulate one or more messenger RNA transcript and, conversely, a given mRNA can be regulated by more than one miRNA [14]. It is possible that miRNA-23b-3p is involved in aortic tissue maintenance; its low expression could be owing to high regulatory activity and catabolism.

Let-7g-3p is the most upregulated miRNA in our study, but didn’t show any direct interaction with the differentially expressed genes in IPA. In the human, the let-7 family is composed of 9 mature let-7 miRNAs highly conserved across different species, suggesting that they may play important roles in the biological processes of various organisms [46]. The tumor-suppressor role of let-7 miRNAs is known [47,48] and the new regulation functions has been described in different clinical situations.

It seems that the let-7 family has an extremely complex regulation network. Koh et. al. predicted that let-7 family miRNAs can regulate 50 genes and affect the downstream target HNF4A, which is a known endodermal differentiation marker [49]. Other study using a porcine shunt model of pulmonary hypertension showed both let-7d-3p and let-7g-3p miRNAs were upregulated, however, let-7f-2-3p was downregulated in endoarterial biopsy samples, suggesting that each let-7 family member has different regulation function [50], suggesting that let-7 family may participate in many molecular pathways and biologic processes simultaneously.

In AAA context, Kin et al. observed that the let-7 family members (let-7a, 7c, 7e and 7f) were significantly upregulated in the AAA tissue [36]. However, there is still no specific information about the association of let-7g and AAA. In this study, even though significant upregulation of let-7g-3p in AAA was observed, no differentially expressed gene showed paired interactions with this miRNA by IPA. Although no interactions between let-7 family and gene expression were found in our analysis, we believe that these miRNAs have an important role in the development of AAA through indirect regulations, which were not a focus of our validation and, consequently, not represented in our IPA network.

A major limitation of this study is the large diameter (5.9 cm) of AAA samples which corresponds to chronic AAA and may involve degeneration process. It is worth mentioning that tissue samples were only possible to obtain during surgery; therefore, the results may not be causal factors, but they might reflect chronic degenerative AAA. Three miRNAs identified in this study were not identified by another group which was searching for circulating miRNAs from patients with an AAA average diameter of 3.6 cm [51]. Probably, a different degree of AAA or biological material origin may have different miRNA profile.

## Conclusions

In conclusion, dysregulation of miRNA-193b-3p, 125b-5p, 150-5p involved in the regulation of genes with a role in the eicosanoid synthesis such as *ALOX5* and *PTGIS*, and metalloproteinase/TIMP pathway, such as *CX3CL1* are potentially implicated in the AAA pathogenesis. The genes and miRNAs identified in this study could be important targets for clinical therapy and diagnosis of AAA.
